# Fruit Eating

**Published:** 1898-02

**Authors:** 


### Fruit Eating.

Each year people grow to appreciate more fully the value of fruit, and eat it, not as a luxury, but as a staple article of food. Fruits are nourishing, refreshing, appetizing and purifying, and consequently have effect upon the health and the complexion. Yet there are differences. Grapes and apples are highly nutritious. Grapes usually agree with the most delicate persons, for they are so easily digested. Nothing is easier to digest than a baked apple, taken either with or without cream. Oranges, lemons and limes are of great value as a means of improving the complexion, and they are especially good if taken before breakfast. Ripe peaches are easy of digestion and are fattening. Nothing is better to enrich the blood than strawberries, which contain a larger percentage of iron than any other fruit. Fruit with firm flesh, like apples, cherries or plums, should be thoroughly masticated, otherwise they are difficult to digest. The skin of raw fruit should never be eaten, and before eating grapes or any small fruit care should be taken to remove all impurities by washing. Never swallow grape stones. Stale fruit and unripe fruit should never be eaten, and very acid fruit should not be taken with farinaceous foods unless the person has vigorous digestion.—*The Sanitarian.*

